# Quantifying social media predictors of violence during the 6 January US Capitol insurrection using Granger causality

**DOI:** 10.1098/rsif.2024.0314

**Published:** 2024-11-06

**Authors:** Qinghua Li, Brayden G King, Brian Uzzi

**Affiliations:** ^1^ Kellogg School of Management, Northwestern University, Evanston, IL, USA; ^2^ Northwestern University Institute on Complex System (NICO), Northwestern University, Evanston, IL, USA; ^3^ McCormick School of Engineering, Northwestern University, Evanston, IL, USA

**Keywords:** collective behaviour, social media, Capitol violence, computational social science

## Abstract

Protests involving brute force are growing in number and are viewed as a likely source of increased collective violence in industrialized nations. Yet, our scientific understanding of how violent protests are related to a leader’s social media communications during protests remains nascent. Here, we analyse new data from the 6 January ‘march on the US Capitol’ to quantify the links between leadership, social media and levels of violence. Using data on thousands of live footage videos, Trump’s tweets and rally speech, other rally speeches and #StopTheSteal tweets, we apply Granger regression methods to analyse the links between former President Trump’s tweets, #StopTheSteal tweets, rally speeches and the severity and duration of outbreaks of violence and weapons use during the riot. We find that Trump’s tweets predict bursts in rioters’ levels and duration of violence and weapons use. Trump’s tweets also predict changes in the volume and sentiments of #StopTheSteal tweets, which in turn explain additional variance in levels of violence and weapons use over the course of the riot. Our findings reveal new patterns of behaviour that link an authority figure’s online behaviour during a protest and the shift from peaceful protesting to violence.

## 1. Introduction

Peaceful protests play a pivotal role in political, legislative and organizational change by shaping law-making agendas and raising awareness about issues [1–4]. Protests can also become violent, leading to property damage and the loss of life. In recent years, there has been an increased incidence of violent protests [5]. For example, far-right protesters clashed violently with counter-protesters in Charlottesville, Virginia, regarding the taking down of a Confederate statue. This ultimately led to the death of Heather Heyer, a peaceful protester. A clash between Black Lives Matter protesters and an armed militia of counter-protesters during a Black Lives Matter rally in Kenosha, Wisconsin, resulted in the death of two protesters and the wounding of a third protester. In 2021, the US officially ranked social movement protests involving brute force and militias as one of the most likely sources of collective violence in the United States [6].

Although attempts to understand the factors associated with the onset of violence during a protest have existed since sociologist Gustave Le Bon first described crowd behaviour in 1895, social media provides a new form of communication and coordination among leaders, activists and the public that raises new questions about how ralliers may radicalize in real time during protests [7–10]. A broad claim in the literature is that the stronger the sentiment expressed in an authority figure’s rhetoric, the more it incites [11,12] and rewards violence [13,14]. Experiments and observational data also suggest that when leaders give voice to grievances, demonstrators feel more authorized to escalate their use of bodily force [15–20]. During the 2015 Baltimore protests, for example, the moral rhetoric used on social media increased on days with violent protests, and the hourly frequency of morally relevant tweets predicted the future frequency of arrests during protests [21]. Posts on VK, the dominant Russian online social network, were found to correlate with violent protests in Russia in 2011, presumably because the posts lowered coordination costs between leaders and followers [22]. These dynamics suggest that a crucial link may exist between the online behaviour of leaders and followers and the real-life actions that can turn peaceful protest into collective violence [23,24].

Recent social psychological research on the 6 January insurrection identifies group-level dynamics behind the uprising, suggesting violence may have emerged from the interaction of protesters, social media communication and the rhetoric of President Trump [25–29]. Some of this research theorizes that previously non-violent protesters at the Capitol were pulled into violent actions due to the escalating rhetoric of President Trump on social media and after observing other protesters engage in violent behaviour [26,27]. Watching their fellow protesters crossing police lines and attacking officers led to an emergent norm that momentarily justified violence [30].

Consistent with these theories, the US Department of Justice and US senators have claimed that social media communications by former President Trump [31] or between the crowd and former President Trump turned the peaceful 6 January ‘march on the Capitol’ violent. Mitch McConnell, the highest-ranking Senate Republican, stated that ‘The people who stormed this building believed they were acting on the wishes and instructions of their president [Trump] … shouting into the largest megaphone on planet Earth [Twitter]’. The 6 January defendants have concurred and attributed their violent actions to Trump’s calls to action. In support of these theories, one recent study found a connection between the offline rhetoric of President Trump and the online rhetoric and memes used by his followers [32]. Nevertheless, the link between a leader’s social media activity and protesting remains debated because of the empirical gap between high-resolution temporal data on online and on-the-ground events during protests [33,34].

Here, we address this empirical gap by examining the unique, high-resolution temporal data of the 6 January ‘march on the Capitol’. This event generated extensive amounts of real-time social media posts and videotaped footage by news agencies and hundreds of persons who were dispersed around the Capitol. At the same time, a full digital record of social media posts was captured in #StopTheSteal tweets, and tweets by former President Trump. Prior to the march on the Capitol, speeches by Rudy Giuliani, Mo Brooks and former President Trump were videotaped along with the crowds at the speech. These data were then made public by news agencies, the US government and public and private websites, creating a corpus of data that permits us to quantify the links between leaderships’ communication on social media and the timing, incidence and duration of violence and weapons used during the violent protest. Our analysis uses Granger causality regression and computational social science techniques to make new measurements and tests [35] of strong, weak and reciprocal predictors of the duration and level of violence and weaponry, as well as the positive feedback loops associated with the duration and escalation of hostility during the Capitol riot.

## 2. Data

After months of false claims about a rigged 2020 presidential election, thousands of pro-Trump protesters assembled in Washington, DC, on 6 January 2021, in support of stopping the certification of the 2020 US Presidential election. After listening to ‘Save America’ rally speeches, the crowd proceeded to the grounds of the US Congress, where hundreds of protesters turned violent. They smashed barricades, vandalized the Capitol and erected a gallows to hang Vice President Mike Pence and Senator Nancy Pelosi for treason. That day, five people died, including one police officer. Over 140 police officers were injured. The hours-long assault ended when former President Trump tweeted for the protesters to go home. In the violence’s aftermath, hundreds of protesters were convicted of insurrection, vandalism, possession of illegal weapons or trespassing.

Our data on the event includes (i) the complete Twitter archive of former President Trump’s (hereafter Trump) 16 tweets, (ii) the complete Twitter archive of 7550 #StoptheSteal tweets [36,37], (iii) the complete text and audio of the 12 rally speech videos [38], and (iv) 1113 time-stamped, live-action videos of the insurrection [31,39] (see electronic supplementary material, table S1 for a list of data sources).

**Table 1.** Tweet sentiment strength coding for Trump’s tweets and #StopTheSteal tweets.

source	time	text	negative sentiment	positive sentiment
Trump	9.00.12	They just happened to find 50 000 ballots late last night. The USA is embarrassed by fools. Our Election Process is worse than that of third world countries!	0.275	0
Trump	1.00.50	If Vice President @Mike_Pence comes through for us, we will win the Presidency. Many States want to decertify the mistake they made in certifying incorrect & even fraudulent numbers in a process NOT approved by their State Legislatures (which it must be). Mike can send it back!	0.149	0.092
Trump	0.43.42	Get smart Republicans. FIGHT! https://t.co/3fs1oPVnAx	0.389	0.29
#STS	13.58.20	So, I've read claims that the #StopTheSteal protestors in D.C. are really #Antifa in disguises.	0.124	0
#STS	12.55.10	You want to #StopTheSteal? There’s only one man who can save America.... https://t.co/5VAzxgttOK	0	0.273

*Trump tweets*. Donald Trump’s complete corpus of 6 January 2021 tweets was archived and posted for public download at https://www.thetrumparchive.com/. We operationalized Trump’s tweets using a variable that measures the length of his tweets, the frequency at which he posts, and the sentiment expressed in those tweets. Tweet sentiment is included in our analysis to capture the possible positive or negative emotions that can motivate extreme action [11,23], including violence [40,41]. Notably, Trump’s 6 January tweets received hundreds of thousands of likes and retweets, indicating that his tweets were read and reacted to [42].

*#StopTheSteal tweets (#STS*). All the tweets with hashtag #StopTheSteal that were posted on 6 January 2021, are publicly available with the search = string ‘#StopTheSteal since:6 January 2021 until 8 January 2021 filter:replies’ (https://twitter.com/search?q=%23StopTheSteal%20since%3A2021-01-06%20until%3A2021-01-08%20-filter%3Areplies&src = typed_query). The following fields are included in the data: Time Stamp, Tweet ID, Text, Username and URL.

Trump and #STS tweets were quantified using VADER [43], a lexicon and rule-based sentiment analysis toolkit designed specifically for social media, including platforms like Twitter, and widely used for its effectiveness in capturing sentiment and emotions expressed in short and informal text. The toolkit calculates the proportion of positive and negative language in each tweet based on tweet’s lexicons, resulting in positive sentiment value (ranging from 0 to 1), neutral sentiment value (ranging from 0 to 1), negative sentiment value (ranging from 0 to 1) and compound sentiment value (ranging from −1 to 1) for each tweet. For our analysis, we constructed two time-series variables of ‘positive sentiment’ and ‘negative sentiment’. We aggregated the sentiment values within each 5 min period over the 24 h of 6 January.

Table 1 provides examples of the coding of Trump and #STS tweets, showing the negative and positive sentiment scores of each tweet. For example, at 13.58 there is a tweet with the handle #StopTheSteal that had the following content, ‘So, I've read claims that the #StopTheSteal protesters in DC are really #Antifa in disguises’. This tweet has a negative sentiment score of 0.124 and a positive sentiment score of 0. Electronic supplementary material, table S3 provides a listing of all of Trump’s 6 January 2021 tweets, their sentiment scores, number of likes and number of retweets.

*Rally speeches*. Trump and others made speeches on the stage of the ‘Save America’ rally at The Ellipse. We use cheer length as an indicator of a crowd’s empowerment and the strength of its collective identity during the speech [44]. Some rally speeches had time gaps between them (the gap to previous/next speech can be 20 s to more than 1 h), and some speeches were contiguous (e.g. Eric Trump welcomed Kimberly Guilfoyle, who in turn welcomed Donald Trump Jr on the stage). We computed the total length of cheers in a speech or connected speeches for each 5 min interval in the speech using CSPAN videos of the speeches. For example, the total length of cheers during Mo Brooks’s warm-up speech is 221 s, which assigned 221 s of cheer to the time interval 9.15 to 9.20. Table 2 shows the rally speaker, start and stop time of each rally speech and the total length of cheers in seconds.

**Table 2.** Save America rally speeches.

speaker(s)	start time	end time	total length of cheers (s)
Mo Brooks	9.06	9.16	221
Katrina Pierson, Amy Kremer	9.41	10.02	303
Vernon Jones	10.05	10.08	91
Ken Paxton	10.09	10.10	19
Eric Trump, Kimberly Guilfoyle, Donald Trump Jr	10.15	10.30	244
Eadison Cawthorn	10.39	10.41	35
Rudy Giuliani, John Eastman	10.48	10:57	164
Donald Trump	12.00	13.11	859

**Figure 1.**
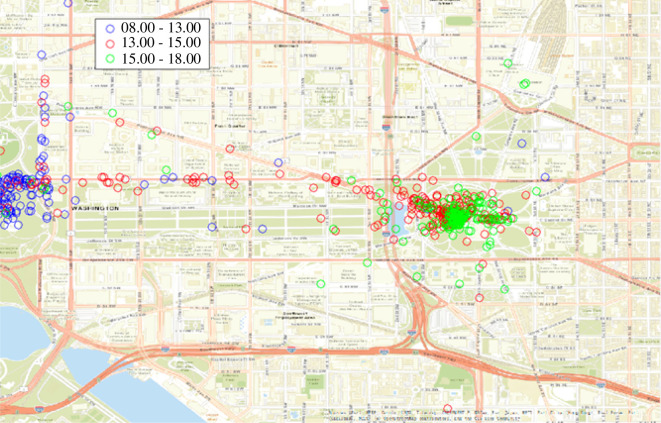
Location of real-time videos by time of day.

*Live action video footage*. We used live-action, real-time videos to quantify the levels of violence and weapons. The videos were uploaded to public websites by protesters, journalists and bystanders who were at different locations during the event. The video archive contains 1113 recorded videos. The average length of a video is 32.80 s (s.d. = 57.60 s; max length = 547 s; min length = 1 s). The extensive footage comprises 150 videos per hour (from 12.00 to 18.00 on 6 January) and 608 min of videotape from the Ellipse, Capitol Hill and Constitution Avenue. All 154 videos were tagged for violence and weapons (see §6.2, table 4). Figure 1 shows the area of activity covered by the videos during 6 January from 8.00 to 18.00 and how video footage follows the movement of the protesters.

To annotate the videos for incidents of violence and weaponry, two research assistants were trained to identify instances of violence and weapons in the videos. If a video showed an instance of violence—say, a beating or a shove—the specific type of violence in the video was tagged and recorded by the assistant. As with our other temporal measurements, we binned the video footage into bins that were 5 min in length and then had trained research assistants tag the video footage for violence and weaponry within each bin. To quantify the severity of violent acts or a weapon’s potential lethality, research assistants used the official District of Columbia’s criminal penalty scale, which offers a standardized point system for quantifying the severity of a violent act and the lethality of weaponry [45]. For example, a beating is more intensely violent than a shove and therefore has a higher penalty (higher point value) than a shove. Specifically, a gunshot = 1500 points; an assault = 1000 points; other violence = 500 points. The severity of the violence or weaponry in a video was the maximum of the video’s separate incidents (e.g. gunshot or nightstick). Table 4 in §6.2 describes the coding procedure’s details.

The Kappa inter-rater reliability scores were 90.63% and 90.28% for violence and weaponry, respectively. Electronic supplementary material, table S2 shows the details of the Washington, DC coding scheme, and electronic supplementary material, figure S1 presents examples of still images and the coding of the identified violence and weaponry. The Washington, DC coding scheme was checked for robustness using an alternative, popular coding scheme based on TV violence ratings [46]. The results under the second coding scheme were confirmatory (electronic supplementary material, table S7).

## 3. Video footage robustness checks

While the social media and rally speech data contain the full contents of Trump’s tweets, all 7550 #StopTheSteal tweets, and the full rally speech text and video, the video on-the-ground footage of the protest is a sample. To address the possible sampling limitations in the video data, we conducted several computational tests typical in the literature on extremism to gauge how robust the results are to measurement error [35,47]. Our tests showed that the results are robust to measurement error and sampling bias.

First, to gauge the stability of our findings to random measurement error, we followed precedent in the literature and randomly removed 5% of the videos and re-ran our analyses on the randomly reduced set of videos [47]. We then compared the significant levels of the full sample findings to the reduced sample findings. We conducted this test 200 separate times, keeping track of the fraction of times the reported (full sample) findings were statistically significant in the reduced sample. Once our fraction was computed, we conducted a binomial test. Our model had eleven statistically significant regression coefficients, and all eleven remained statistically significant at a level unexpected by chance according to the binomial tests. Similarly, all the variables that were non-significant in our model remained non-significant, according to the binomial test.

Second, we conducted the above test again but in the opposite direction. This time, we added 5% more violence to the videos at random to detect whether adding mismeasured violence would change the reported results. These tests further indicate that the reported results are unlikely to be due to measurement errors in the coding or in the sampling of the videos.

**Figure 2.**
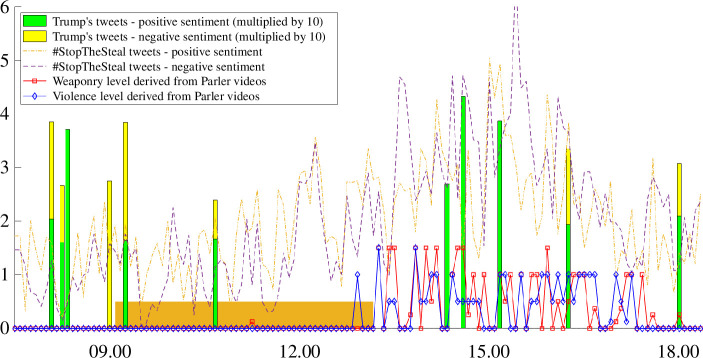
Time-series of events. Figure plots the time-series of variables used in the analysis. The period during which the ‘Save America’ rally took place is shaded in orange.

Figure 2 is an ensemble plot of the time-series of each data source—sentiment strength of Trump’s tweets, sentiment strength of #StopTheSteal tweets and Parler video-derived variables of weaponry level and violence level. The period of the ‘Save America’ rally speeches is shaded in orange. Variables are sampled at 5 min intervals.

*Granger modelling*. Granger regression modelling is a widely used and validated model that infers a predictor variable’s ‘Granger causality’ relationship with an outcome variable in time-series data [48–55]. Granger regression tests are appropriate for our analysis because we are interested in testing one-way and reciprocal relationships, namely, that time-series *X* (i.e. Trump’s tweets) predicts changes in time-series Y (i.e. level of violence) and vice versa. Many analyses use Granger regression to infer causality in observation data. We present and interpret the Granger results conservatively as a test of whether a significant correlation exists between *X* and *Y* variables [52]. Formally, after passing the stationarity test, a time-series of *X* is considered to ‘Granger predict’ Y if *t*-tests and *F*-tests on lagged values of the *X* variables and lagged values of *Y* significantly predict future values of *Y*.

To meet the data requirements for a Granger analysis, we followed prior research [56–62]. First, the time-series data must be stationary. Accordingly, we conducted standard Dicky–Fuller stationarity tests, and all variables passed successfully with up to two lags (i.e. a total lag of 10 min). Table 3 shows that all stationarity tests for lags of one and two passed the Dicky–Fuller tests. Second, it is important for the Granger data not to exhibit seasonality. In our case, the data shows no evidence of seasonality. Third, the presence of zeros in the Granger data is permissible. In our dataset, zeros are indeed present. Thus, our Granger analysis aligns with established precedents in economics and neuroscience that use stationary, non-seasonal data that can contain zero values [56–62].

**Table 3.** Dicky–Fuller test results for stationarity.

variable	lag = 1 (5 min)	lag = 2 (10 min)
violence level derived from Parler videos	−7.164, (*p* = 0.0000)	−5.091, (*p* = 0.0000)
weaponry level derived from Parler videos	−6.305, (*p* = 0.0000)	−5.269, (*p* = 0.0000)
negative sentiment of Trump’s tweets	−11.730, (*p* = 0.0000)	−8.322, (*p* = 0.0000)
positive sentiment of Trump’s tweets	−11.660, (*p* = 0.0000)	−7.806, (*p* = 0.0000)
positive sentiment of #STS tweets	−5.191, (*p* = 0.0000)	−3.975, (*p* = 0.0015)
negative sentiment of #STS tweets	−4.087, (*p* = 0.0010)	−3.309, (*p* = 0.0145)
cheer length—Trump’s speech	−11.958, (*p* = 0.0000)	−9.747, (*p* = 0.0000)
cheer length—other speeches	−10.872, (*p* = 0.0000)	−9.107, (*p* = 0.0000)

Granger tests regress outcome variable *Y* on its own lagged values and the lagged values of predictor variables *X*. The null hypothesis test is that the estimated coefficients on the lagged values of *X* are jointly zero. Our findings were obtained from the Stata commands *var* and *vargranger*. The *vargranger* command indicates the Granger bivariate relationship between each *Y* and each *X,* net of the effects of other *X*s in the same equation [63]. Thus, depending on the equation, the variable violence level is both an outcome variable and a predictor variable. This rotating set of outcome variables allows reciprocal (or bidirectional) Granger tests among the variables in the model. Our model takes eight time-series variables: violence level, weaponry level, negative and positive sentiment of Trump’s tweets, negative and positive sentiment of #StopTheSteal tweets, Trump’s speech cheers and warm-up speech cheers. Thus, there are eight models; and each model has a different dependent variable that is regressed on 14 independent variables (seven variables ∗ two lags).

A robustness check of the Granger model was performed with MVGC Matlab^®^ Toolbox (electronic supplementary material, table S5), which produced confirmatory results. Further, for each equation and each endogenous variable that is not the outcome variable of the equation, we conducted Wald tests, which test the hypothesis that each of the other endogenous variables does not predict the outcome variable of that equation.

Formally, our model is written as


$$\;\;X_{i,\;t}=\sum_{j=1}^{J}\sum_{\tau =1}^{L}a_{j,r}\cdot X_{j,t-\tau }+\epsilon_{j,t}$$

where $X\left(t\right)\in R^{J\times 1}$ for *t* = 1, …, *T* is a *J*-dimensional multivariate time-series. For each equation, *vargranger* reports Wald tests on the hypothesis the other endogenous variables are unrelated to the dependent variable in that equation.

## 4. Results

**Figure 3.**
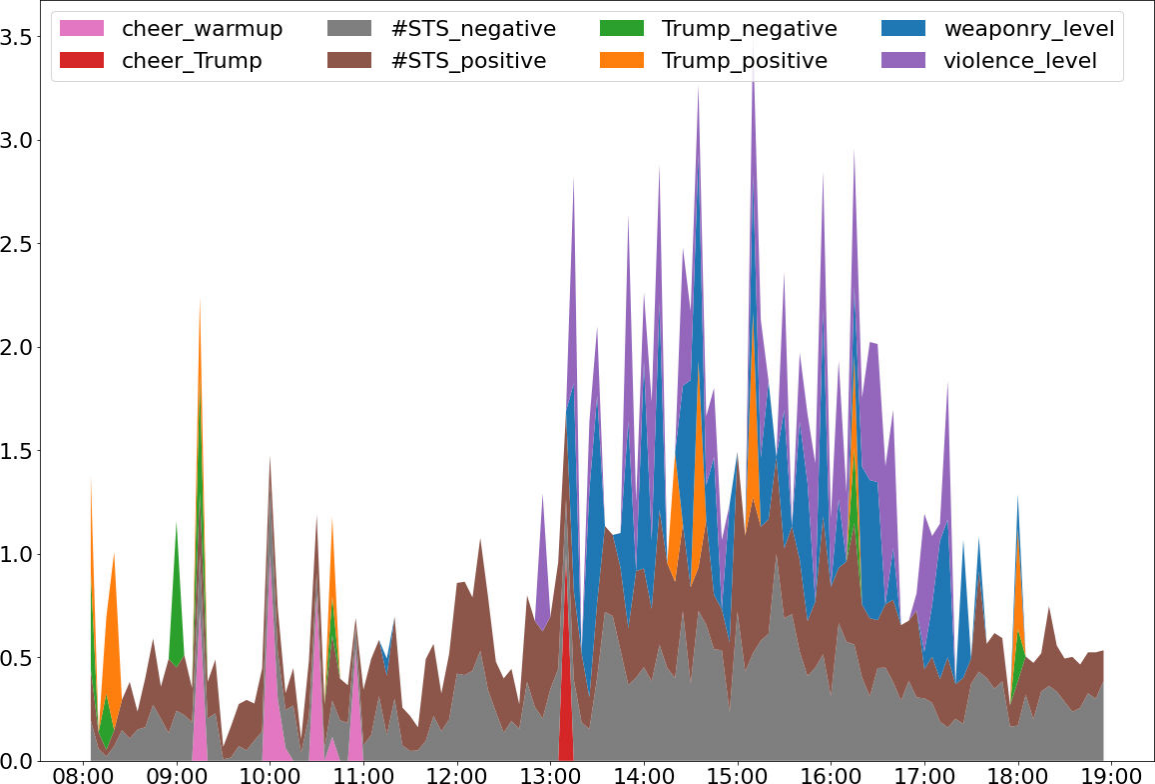
Steam plot of the chronology, co-occurrence, and magnitude of variables in the analysis. Variables are normalized by their maximum value to permit comparisons across different measurement units and are stacked so that the normalized magnitude of a variable at any point is the magnitude of the variable shown on the *y*-axis less the sum of the magnitudes of the variables stacked below it. In the figure the escalation of rioting follows immediately after Trump’s rally speech, mounts as Trump and #StopTheSteal tweets increase and de-escalates after Trump stopped tweeting and the frequency of #StopTheSteal tweets waned.

Figure 3 provides a discriptive visual analysis of the chronology of variables and their intensity, normalized by a variable’s maximum value to permit comparisons across different measurement units. The variables in the figure are stacked to make all variables visible, such that the normalized magnitude of a variable at any point is the magnitude of the variable shown on the *y*-axis, less the sum of the magnitudes of the variables stacked below it.

The figure provides several descriptive findings. First, variables based on the actions of specific individuals in positions of leadership or authority, such as Trump and the speechmakers, occurred at specific times during the riot and hence appeared as spikes (e.g. Trump’s negative tweets at 8.15 and 9.00). The densest activity occurred from 13.10 to 17.10, with peaks at 13.10, 14.20, 14.35 and 15.10. During these dense clusters of activity, the highest frequency and highest levels of violence (purple spikes) and weaponry (blue spikes) took place. Also, at the beginning of these dense clusters of activity, we observe the highest positive sentiment in Trump’s tweets. While violence and weaponry levels co-occur in these dense clusters of activity, spikes in violence tend to precede spikes in weaponry levels. Finally, we observe that the escalation from peaceful protest to violent attacks and weapons use initiates after Trump’s 11.00 rally speech and continues to mount as the frequency of Trump’s and #StopTheSteal tweets increase. Rioting peaks at around 15.30 and then begins to de-escalate over the next 2 h, during which time Trump also stops tweeting. The rioters finally cleared the Capitol grounds after Trump provides a video asking them to leave.

**Figure 4.**
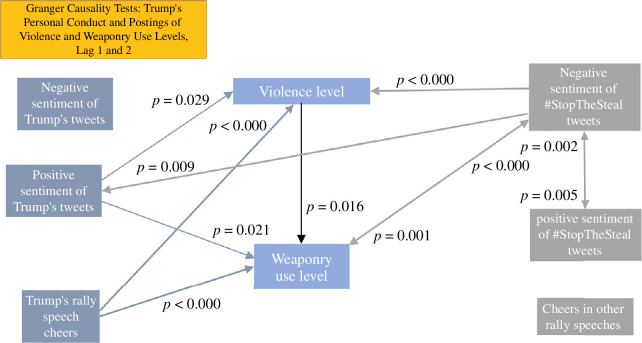
Granger regression estimates of the strength of Trump’s tweets, #StopTheSteal tweets and rally speeches in predicting levels of violence and weapons during the Capitol insurrection. The figure shows the ‘Granger-causal’ predictors of violence and weaponry during the 6 January insurrection. Arrowheads indicate the direction of effects, bold and normal arrows represent the statistical significance of the effects (exact *p*-values shown above arrows), and the colours of boxes represent measures of violence and weapon’s use (blue boxes), Trump’s conduct (blue–grey boxes) and the crowd’s conduct (grey boxes). The thickest arrows represent the strongest statistical relationships that can be expected to happen by changes less than 1% of the time.

Figure 4 shows the Granger estimates of the strength and direction of relationships among predictor variables and levels of violence and weaponry during the 6 January protest. In the figure, arrowheads indicate the direction of associations and their statistical significance (exact *p*-values shown above arrows). Blue–grey boxes represent Trump’s tweets and rally speech cheer variables, blue boxes represent violence and weaponry variables, and light grey boxes represent #StopTheSteal tweets variables and the rally speech cheer variables of the other rally speakers. A non-statistically significant relationship in the model is represented by an absence of a link (see electronic supplementary material, table S4 for full regression output and Wald tests).

**Table 4.** Coding procedures for Parler video dataset.

ID	question	data type	question type
1	Record the video ID.	string (url)	time-series building
2	Enter the length of the video.	seconds	quality control
3	Is there physical violence in the video?	boolean	time-series building
4	Please record violent acts and when they occur.	string	time-series building
5	Are there weapons in the video?	boolean	time-series building
6	If weapons are present, please select any that appear from the list below and when they are present.	string	time-series building
7	Please describe anything else that you find noteworthy in the video.	string	quality control

The Granger mapping provides a systemic, multivariate perspective on the dynamics of the 6 January insurrection and indicates how Trump’s behaviour and the protesters’ behaviour interdependently led to an escalation in violence and weapons use. First, Trump’s tweets and rally speech have four important connections in the model. Trump’s tweets significantly predict levels of violence (*p* < 0.029) and use of weapons (*p* < 0.021) during the insurrection. Second, Trump’s rally speech cheers predict the subsequent use of violence (*p* < 0.000) and use of weapons during the march (*p* < 0.000). None of the other rally speeches show a statistically meaningful relationship with any other variable in the model.

Third, #StopTheSteal tweets directly predict violence and weaponry use. Negative sentiment #StopTheSteal is positively related to violence levels ((*p* < 0.000) and weaponry use (*p* < 0.001) with more weaponry use having a reciprocal relationship with negative sentiment #StopTheSteal tweets (*p* < 0.000) and an indirect relationship with positive #StopTheSteal tweets through negative #StopTheSteal tweets (*p* < 0.005). Fourth, increases in violence predict an increase in the use of weaponry (*p* < 0.016). From this systemic perspective, the results suggest that the 6 January ‘march on the Capitol’ feeds on itself through positive feedback loops among predictor and outcomes variables that quickly escalate peaceful protesting violence and weapons use.

**Figure 5.**
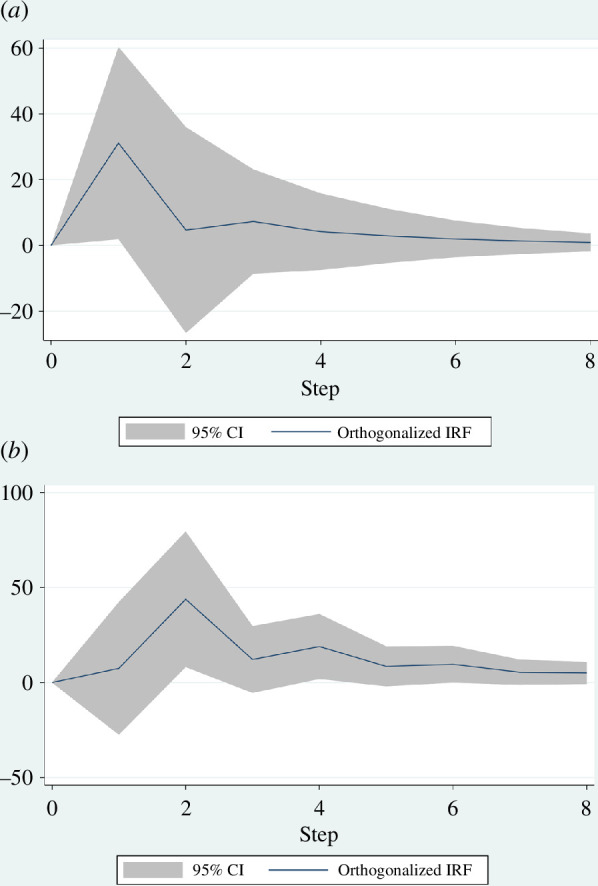
Impulse response analysis. Plots of orthogonalized impulse response functions (IRF) show the estimated magnitude and duration of changes in the violence level (*a*) and weaponry level (*b*) of the crowd in response to the sentiment of Trump’s tweets. The *x*-axis represents time steps that are 5 min in duration.

Further examination of the Granger regression estimates permits estimates of the magnitude and duration of effects using the Stata vector autoregressive models (VAR) command. Figure 5 shows the impulse responses of Trump’s tweets in relation to changes in the level of violence and weaponry. In VAR like Granger regression, the impulse response quantifies the change in one variable(s) per changes in other variable(s). In systems like the Capitol protest that have stationary data, it is expected that the response of one variable to a change in another variable is impermanent: the variable’s response dissipates over time as the system reconverges. Methodologically, after the VAR is solved, the impulse response can be calculated by setting the input of the model as a unit impulse of *x*(*t* = 0) = 1, *x*(*t* = 1, 2, …) = 0.

Figure 5*a,b* quantifies the magnitude and length of the change in violence (figure 5*a*) and weaponry (figure 5*b*) per a change in the sentiment strength of Trump’s tweets. The magnitude of change for the response variable is shown on the *y*-axis. The duration of the response is shown on the *x*-axis and is determined by the length of the period for which the stardard error (SE) bands are above 0.

A time step in our data represents a 5 min interval. The figures indicate a substantial response relationship between the sentiment strength of Trump’s tweets and the magnitude and duration of violence and weaponry levels. A one-unit change in sentiment strength of Trump’s tweets leads to a substantial increase in both the magnitude and duration of violence and weaponry. Specifically, for violence level, the response shows a significant increase of 30 units, which lasts for two time periods. Similarly, weaponry level exhibits a notable increase of 50 units, which persists for three time periods. The effects gradually dissipate after these periods. The bursty nature of the impulse responses suggests that the relationship between a leader’s and protesters’ behaviours is strongest when they are continuous.

## 5. Discussion

The 6 January Capitol insurrection provides a distinctly informative case study of collective violence. Our analysis found new evidence of direct, indirect and reciprocal predictors of the duration and level of violence and weaponry use. The findings are broadly supportive of claims that social media can provide an instantaneous means of communication that is directly associated with subsequent on-the-ground actions, including violence. In our data, we observe that emotive rhetoric on social media during a protest is strongly correlated in real time with the radicalization of an already aggrieved crowd. Our analysis also points to the importance of a leader in escalating violent protest behaviour. Trump’s rally speech and his tweets predict violence and weapon use levels as well as #StopTheSteal tweets, which in turn further explain changes in the levels of violence and weapon use.

Our findings have implications for the broader debate about whether social media’s polarizing effects [64] and ability to spread misinformation [65–67] at an unprecedented scale enables leaders to manipulate supporters’ real-life violence [68]. Prior work shows that increases in the number of tweets with moralized language predict later numbers of protester arrests during instances of civil disobedience but not outright violence [21]. Using real-time data linking social media posts by a protest’s leader and protesters predict the shift from a peaceful protest into one teaming with the use of violence and lethal weapons.

Our analysis supports other research which has argued that there is a dynamic relationship among leaders and protesters, viz., leaders and followers can escalate each other’s support for more confrontational and bloodthirsty tactics through emotive rhetoric that can incite action and the use of bodily force [11,13,14,69]. First, our results support these theoretical expectations and suggest that the positive reciprocal relationship between leaders and followers can quickly intensify, changing a peaceful protest into a violent attack that builds on itself. Whereas past research has suggested that violence at the Capitol emerged from Trump’s escalating rhetoric on social media and observing fellow protesters’ aggression, our study provides empirical evidence of this reciprocal process that ultimately led to violent actions against police officers [30].

Second, theories of collective behaviour tend to emphasize group processes that lead to emergent, potentially violent collective action, whereas social movement theories usually focus on the stabilizing organizational elements of collective action, including the strategic role of the leader [25,70,71]. Our paper bridges these approaches by showing how the strategic rhetoric of a leader can trigger group processes that ultimately led to the violent insurrection on 6 January. Rather than being a moderating force, our study demonstrates that movement leadership can also escalate protests into violence, especially when amplified through social media. Our study, thereby, shows the continued relevance of social psychological theories of group behaviour to explaining protest violence on 6 January specifically [25–28], but we believe it has broader implications for understanding the escalation of violence or violent rhetoric in contemporary politics [30]. In particular, we show that leaders are not always stabilizing forces but also have the ability to trigger protesters into taking violent actions.

Third, our findings have implications for the broader debate about whether social media’s polarizing effects [64] and ability to spread misinformation [65–67] at an unprecedented scale enables leaders to manipulate supporters’ real-life violence [68]. Prior work shows that increases in the number of tweets with moralized language predict later numbers of protester arrests during instances of civil disobedience but not outright violence [21]. Using real-time data linking social media posts by a protest’s leader and protesters predicts the shift from a peaceful protest into one teaming with the use of violence and lethal weapons. Our analysis supports the perspective that leaders have gained new power in influencing their followers through social media; more specifically, our study indicates that social media is more than a passive communication channel. Social media companies can both enlarge or diminish a leader’s impact on a crowd by regulating communication and content in a system of reciprocal feedback loops that can rapidly escalate violent confrontations. In our study, protest leaders were associated with the onset of these escalation dynamics. Yet, leaders’ communications are linked to short-term bursts of violence and other activities that tend to peter out unless new communications are put forth. This suggests that reducing leaders’ communications in real time could possibly begin de-escalation. Together, these findings suggest that future research on social movements and violent behaviour would benefit from an understanding of the factors that create and sustain feedback loops, including social media companies that control how algorithms push information to users [72].

Fourth, besides the potential implications of the 6 January ‘march on the Capitol’ for current theories of how protests can turn violent, it raises new theoretical questions about how the consequences of a single case can play out in time long after the protest and violence ends. The 6 January ‘march on the Capitol’ case demonstrates that a single extreme act that goes further than had previously been imagined can result in far-reaching and long-term policy changes as well as become a model for future threats to democracy as news about the event rapidly spreads across the globe [73]. As other research on 6 January has stated, although this may be one incident of political violence, it will certainly not be the last [25]. Scholars and policymakers alike need to be more attuned to the role that political leaders and social media play in escalating violence and identifying new procedures for shoring up democracy. Future research should begin to study, therefore how social media aids not just in a leader’s impact during a protest but on how social media spreads protest tactics well beyond the boundaries of a single event.

The case also presents limitations. Like any single case, generalizing findings must be done cautiously. This case, for example, is within the context of a society where deadly weapon ownership is permitted and, therefore, allowed many protesters to bear and conceal arms at the protest that were later used violently. Similarly, the US has election procedures that created a particular context for this case. Finally, as a single case, it is unclear how variation in the personalized relationships between a protest leader and their followers might change the dynamics we observed in the 6 January ‘march on the Capitol’ case. While it is reasonable to hypothesize that our findings are likely to generalize depending on the strength of the personal relationship between the leader and their followers, future research is needed to know whether that conjecture is supported by test data.

The 6 January insurrection had a significant impact on American politics, security practices and public discourse, leaving a lasting imprint on the nation’s history. It intensified polarization within the United States; individuals involved in the insurrection have been arrested and sentenced for various crimes, and changes in the interpretation of the constitutions protections for presidential acts followed. Law enforcement investigations continue to identify and prosecute perpetrators years after the event, and future regulation on social media has been called for, even as some platforms are taking stricter measures against extremist views and other platforms are encouraging more extreme views. Future research should continue to examine this case and its aftermath to begin to develop a richer understanding of the new links between social media, leaders and followers.

## 6. Material and methods

### 6.1. Materials

All data used in the study was either available for public use per its terms of service or received written permission for its use by its owner. The Insitutional Review Board (IRB) approved this study (STU00216418).

### 6.2. Methods

#### 6.2.1. Coding of videos for violence and weapons

Two trained assistants coded the 6 January videos for instances of violence and weaponry. The assistants watched, analysed and then coded Parler videos by responding to the seven questions outlined in table 4. The inter-rater reliability score of the two coders was high to moderate with a Kappa of 90.63% and 90.28% for violence and weaponry, respectively.

Of the questions listed in table 4, we employ four to construct time-series variables related to violence level and weaponry level. Additionally, we use two questions to assess and ensure the quality of the responses. Specifically, ‘weapon’ encompasses guns, clubs, batons, etc., and ‘violence’ includes shooting, pushing, etc., following the agreement of two popular scales, the DC legal status (electronic supplementary material, table S2) as well as the TV violence scale as a robustness check (electronic supplementary material, table S6).

### 6.3. Operationalizing video events as a time-series dataset

We built the time-series in 5 min intervals according to the responses in the fields 1, 3, 4, 5 and 6. The timestamp of each video was extracted from its meta information. With the sorted timestamps, we created a variable ‘violence level’ based on the answers to questions 3 and 4, and variable ‘weaponry level’ based on the answers to questions 5 and 6. ‘Violence level’ and ‘weaponry level’ are continuous variables and were constructed based on the statutory criminal punishments associated with a certain level of violence or a certain weapon. Statutory penalties are designed to be proportional to the severity of the crime (source: https://koehlerlaw.net/criminal-defense-dc/weapons/ and https://criminallawdc.com/dc-assault-lawyer/laws/).

Electronic supplementary material, table S2 lists the criminal penalties for different violent acts or weapons. Penalties vary according to DC statutes. For example, the penalty associated with ‘weapons’ is a ‘maximum fine of $1000.’ Given that firearms are considered deadly or dangerous weapons, we assign a value of 1500 points to firearms and a value of 1000 points to clubs/bats/etc., for the violence and weaponry variables. Unconventional objects used as weapons, such as extinguishers, canes and flagpoles were assigned the lowest value of 500. The violence level and weaponry level for each 5 min interval is calculated by taking the maximum of the scores of all videos taken during that interval. The mean (s.d.) of violence level is 116.319 (311.528), and of weaponry is 147.569 (398.072).

### 6.4. Granger regression analysis

#### 6.4.1. Wald tests

The Wald test investigates the hypothesis that each of the other endogenous variables does not Granger-predict the dependent variable in that equation. Using a standard convention for reporting the regression results of Granger analysis, the column ‘Equation**’** represents the *Y* variable of the regression, and the column ‘Excluded’ represents the *X* variables of the regression. We set the critical value of our hypothesis test at the 10% level with lags = 2. All the bivariate relationships with *p* ≤ 0.05 are reported in the figure; otherwise, the link is omitted. The full regression results are shown in electronic supplementary material, table S4.

#### 6.4.2. Multivariate Granger test in Matlab^®^ toolbox: robustness check

To test whether the Granger relationships reported by the STATA Granger algorithm are method dependent, we ran Granger analyses with MVGC multivariate Granger test in the Matlab toolbox, which conducts numerical computation and statistical inference of Granger relationships. The two approaches return the same statistically significant Granger results. The full regression results are shown in electronic supplementary material, table S5.

#### 6.4.3. Alternative coding scheme for violence

We conducted multiple Granger tests with alternative coding schemes for violence and weaponry levels to test whether the results were coding dependent. For the alternative variable *violencelevel*, the coding method is derived from a rating for TV violence [46] and summarized in electronic supplementary material, table S6. The alternative rating scheme produced confirmatory results.

## Data Availability

Data and codes are available in a Zenodo repository [74]. Supplementary material is available online [75].
